# Single‐cell high‐content imaging parameters predict functional phenotype of cultured human bone marrow stromal stem cells

**DOI:** 10.1002/sctm.19-0171

**Published:** 2019-11-23

**Authors:** Justyna M. Kowal, Hagen Schmal, Ulrich Halekoh, Jacob B. Hjelmborg, Moustapha Kassem

**Affiliations:** ^1^ Department of Endocrinology and Metabolism, Molecular Endocrinology Laboratory (KMEB), Odense University Hospital University of Southern Denmark Odense Denmark; ^2^ Department of Orthopedics and Traumatology Odense University Hospital Odense Denmark; ^3^ Department of Epidemiology, Biostatistics and Biodemography Odense University Hospital Odense Denmark; ^4^ Department of Cellular and Molecular Medicine, Danish Stem Cell Center (DanStem) University of Copenhagen Copenhagen Denmark; ^5^ Stem Cell Unit, Faculty of Medicine King Saud University Riyadh KSA

**Keywords:** cell and nucleus morphology, high‐content imaging, human stromal/mesenchymal stem cells, osteoblastic and adipocytic differentiation, proliferation

## Abstract

Cultured human bone marrow stromal (mesenchymal) stem cells (hBM‐MSCs) are heterogenous cell populations exhibiting variable biological properties. Quantitative high‐content imaging technology allows identification of morphological markers at a single cell resolution that are determinant for cellular functions. We determined the morphological characteristics of cultured primary hBM‐MSCs and examined their predictive value for hBM‐MSC functionality. BM‐MSCs were isolated from 56 donors and characterized for their proliferative and differentiation potential. We correlated these data with cellular and nuclear morphological features determined by Operetta; a high‐content imaging system. Cell area, cell geometry, and nucleus geometry of cultured hBM‐MSCs exhibited significant correlation with expression of hBM‐MSC membrane markers: ALP, CD146, and CD271. Proliferation capacity correlated negatively with cell and nucleus area and positively with cytoskeleton texture features. In addition, in vitro differentiation to osteoblasts as well as in vivo heterotopic bone formation was associated with decreased ratio of nucleus width to length. Multivariable analysis applying a stability selection procedure identified nuclear geometry and texture as predictors for hBM‐MSCs differentiation potential to osteoblasts or adipocytes. Our data demonstrate that by employing a limited number of cell morphological characteristics, it is possible to predict the functional phenotype of cultured hBM‐MSCs and thus can be used as a screening test for “quality” of hBM‐MSCs prior their use in clinical protocols.

## Graphical abstract

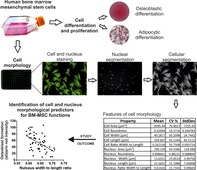

**Significance statement**

Clinical trials employing cultured human bone marrow mesenchymal stem cells (hBM‐MSCs) demonstrate the presence of a large inter‐donor variability in biological functions and clinical efficacy. By applying high‐content imaging methodology to cultured BM‐MSCs obtained from a large cohort of donors, morphological features including cell area and nucleus geometry as predictors for cell differentiation and proliferation were identified. These parameters can serve as selection criteria for BM‐MSC populations to be used in clinical trials of enhancing bone regeneration.

## 1 INTRODUCTION

Human bone marrow‐derived stromal (mesenchymal) stem cells (hBM‐MSCs) are non‐hematopoietic, self‐renewing, plastic adherent cells with the ability to differentiate into several mesodermal lineages including osteoblasts (OB) and adipocytes (AD).^1^ The efficacy of hBM‐MSCs transplantation to enhance skeletal and non‐skeletal tissue regeneration, for example, following bone fracture, cartilage injury, as well as cardiovascular and immune diseases, is being examined in a large number of clinical trials. The rationale is that hBM‐MSCs can differentiate into functional bone forming osteoblastic cells (skeletal applications) or produce a large number of cytokines and growth factors (so‐called cytokine factory) that improve tissue regeneration (non‐skeletal applications).^2^, ^3^, ^4^ Although MSCs‐like cells have been isolated from different tissues such as adipose tissues, placenta, and Wharton's jelly of umbilical cord, bone marrow is considered the standard source for MSCs with in vitro and in vivo bone‐forming ability^1^, ^5^, ^6^ and is the basis for their clinical use for enhancing bone tissue formation.

The reported clinical outcome of hBM‐MSC‐based therapies for treating non‐healed bone fractures or bone defects has revealed inconsistent results with respect to the efficacy of in vivo bone formation, which may be explained by variations in the phenotype of the transplanted cells.^1^, ^7^ In vitro cultured hBM‐MSCs exhibit cellular heterogeneity with respect to their potential for osteoblast differentiation and bone formation.^8^, ^9^, ^10^ Furthermore, cultured hBM‐MSCs demonstrate inter donor variations related to donor age, sex, or disease state.^11^, ^12^, ^13^

Comparing the outcome of clinical trials for bone regeneration that has used hBM‐MSCs is often hampered by the lack of “common” cellular biomarkers that define the “quality” of transplanted cells. Identification of biomarkers that facilitate the selection of clinically relevant hBM‐MSC populations is needed in order to improve the outcome and consistency of hBM‐MSC‐based therapies. Traditionally, hBM‐MSCs have been defined by a limited number of CD markers, for example, CD44, CD73, CD90.^1^, ^5^, ^6^ These markers are sensitive but not predictive of the differentiation capacity of the cells.^9^, ^14^ Several studies have identified a molecular signature for bone‐forming hBM‐MSCs by applying global analysis of gene expression,^9^, ^15^, ^16^ miRNA expression,^17^, ^18^ or proteome analysis.^19^, ^20^ Although these approaches contribute significantly to understanding the biology and functions of hBM‐MSCs, they are labor‐intensive, use a large number of cells, and may not be easy to implement in a clinical setting.

Changes of cell morphology have been observed during cell proliferation^21^, ^22^ and differentiation^23^, ^24^ and thus are determinant of the biological functions of the cells. The relationship between cell morphology and biological functions have been studied in respect to changes in cytoskeletal fibers (actin and tubulin) that mediate cellular adaptation to microenvironmental stimuli and facilitate intracellular signal transduction. For example, the actin cytoskeleton plays a role in hBM‐MSC lineage commitment,^25^, ^26^ and microtubules are key players in cell proliferation^27^ and have been reported to contribute to osteoblast differentiation.^28^ Alterations in cell shape affects actin stress fibers, which through intracellular signaling pathways initiate cell lineage commitment.^23^, ^29^ Moreover, nucleus shape can be modulated by cytoskeletal fibers in response to extracellular forces that affect cellular differentiation.^30^ Liu et al showed that culturing cells on surface with different topography could affect nuclear morphology and cell differentiation in rat BM‐MSCs. Cells cultured on high micropillars exhibited altered nucleus shape that favored osteoblastic differentiation, whereas cells grown on smooth or on low micropillars exhibited less deformed nuclei which were associated with adipocyte differentiation.^24^

The availability of quantitative high‐content imaging technologies at a single‐cell resolution has allowed studies on the role of cell morphology as a predictor of cell differentiation potency.^31^, ^32^, ^33^ However, these studies did not investigate the morphology of undifferentiated hBM‐MSCs and focused on cells at an early stage of differentiation. We applied high‐content screening (HCS) technology at a single‐cell resolution of a large cohort of primary hBM‐MSCs cultured under conditions relevant to the clinical use of the cells. Our aim was to identify a set of morphological features in primary hBM‐MSCs, to serve as predictors for the biological functions of hBM‐MSCs. We used correlation analysis of morphological parameters to the degree of cell proliferation and differentiation capacity into osteoblastic versus adipocytic cells as the primary outcome.

## 2 MATERIALS AND METHODS

### 2.1 Donors and materials

Bone marrow aspirates were collected from lower extremities of 56 adult donors: 26 males (age 18–81 years) and 30 females (26–97 years), undergoing surgeries at the Department of Orthopedics and Traumatology, Odense University Hospital. The samples of bone marrow were considered as “waste material” from routine operations, and, therefore, the material collection was without extra risk for patients. All subjects received oral and written information and signed a consent. The project was approved by Scientific Ethics Committee of Southern Denmark (project ID: S‐20160084).

### 2.2 Cell isolation and culture

Bone marrow (5–10 mL) was collected into EDTA‐coated vacutainers. hBM‐MSCs were cultured from mononuclear cell population following gradient centrifugation using lymphoprep of the bone marrow, through plastic adherence as described previously.^13^ The cells were cultured in MEM media supplemented with 10% fetal bovine serum (FBS; the same lot number 42F0266K was used for all samples) and 1% penicillin/streptomycin (P/S), in standard culture conditions (37°C in humidified 5% CO_2_ incubator). After a week, when the first adherent cells were visible, media was switched to MEM media supplemented with 10% FBS, 1% P/S, 1% GlutaMAX, 1% sodium pyruvate, and 1% nonessential amino acids (S‐MEM growing medium). When the cells reached around 80% of confluence, they were trypsinized and used for analysis and for further cell expansion.

### 2.3 Study workflow

**Figure 1:**
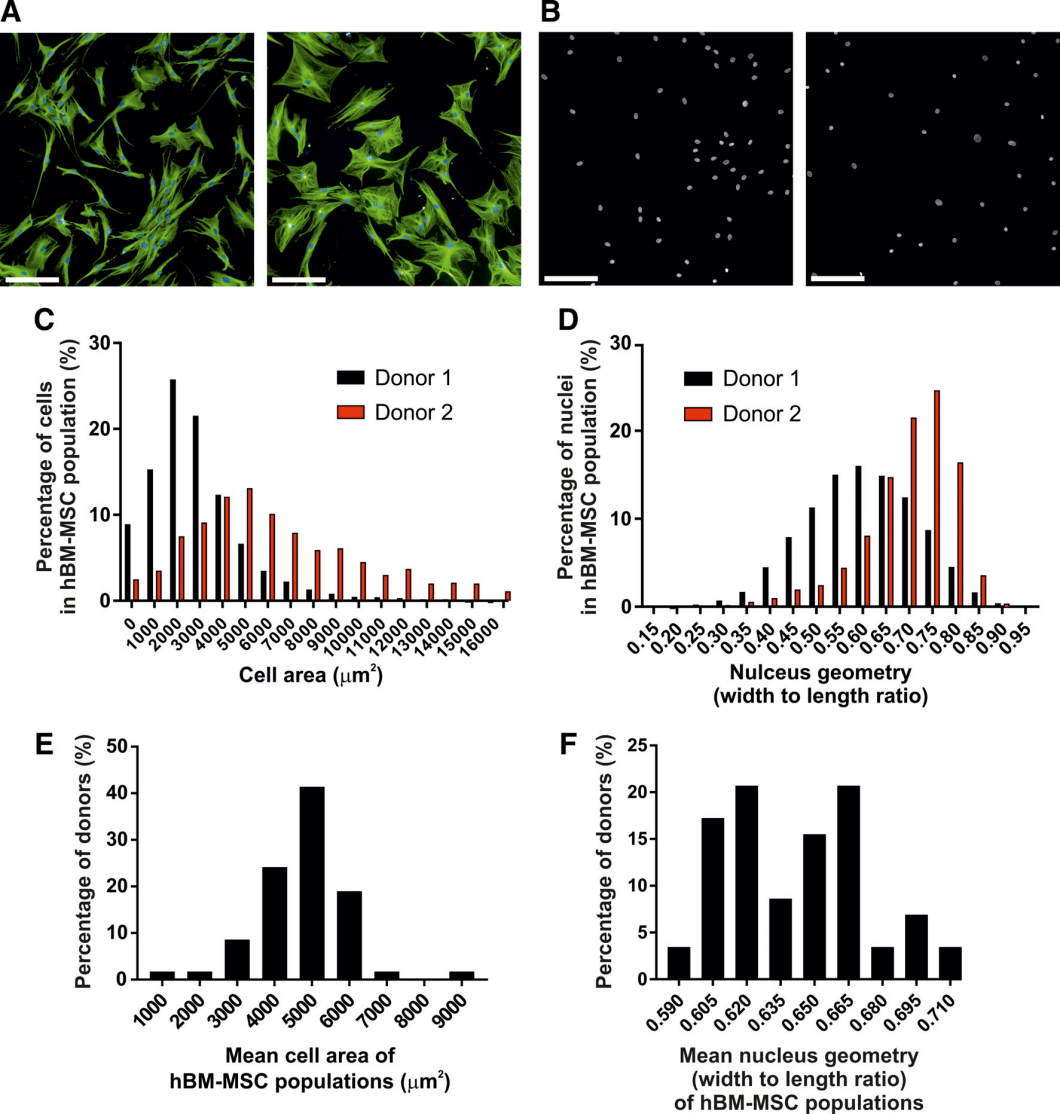
Variations in cell and nucleus morphology of cultured hBM‐MSCs. A, Representative photomicrographs of cultured hBM‐MSCs with contrasting cell morphology. Left photomicrograph: cultured cells from donor #1 exhibit more fusiform and smaller size compared with donor #2 cells that are large cuboidal cells (right photomicrograph). Scale bar is 200 μm. B, Representative photomicrographs illustrating heterogeneity of nucleus morphology. Donor #1 (left photomicrograph) cultured cells exhibit more oval shaped nuclei compared with donor #2 cells that exhibit more rounded shaped nuclei (right photomicrograph). Scale bar is 200 μm. C, Quantitative frequency distribution of cell areas of cultured hBM‐MSCs derived from donor #1 and donor #2. D, Quantitative frequency distribution of nucleus shapes expressed as nucleus width to length ratio of cultured hBM‐MSCs derived from donor #1 and donor #2. Quantitative frequency distribution of mean cell areas (E) and nucleus shape (F) of cultured hBM‐MSCs derived from all donors (n = 56). hBM‐MSCs, Human bone marrow‐derived stromal (mesenchymal) stem cells

The study workflow is illustrated in Figure S1. The cells were trypsinized (1) and prepared for osteoblastic and adipogenic differentiation, cell proliferation, cell membrane marker expression, and morphology analysis. Cell morphology was studied on whole populations of primary hBM‐MSCs obtained from each donor and at single‐cell resolution (2). After culturing hBM‐MSCs in standard medium for 48 hours, the cells were fixed, stained for cytoskeletal fibers, and visualized using automated fluorescence microscope (Operetta HCS) (Perkin Elmer). After nuclear and cellular segmentation (3), the basic cell and nucleus morphology parameters as well as cytoskeletal texture patterns were analyzed (4). The table in Figure 1 lists cell morphology features observed in the representative photomicrograph (5) and provides mean values and coefficient of variations and standard deviation (SD) of each parameter. All the quantified cellular and nuclear morphological features were correlated with corresponding in vitro assays described in the following paragraphs.

### 2.4 Cell proliferation

Short‐term proliferation was performed in cultured hBM‐MSCs at first passage. The cells were seeded in a 6‐well plate (1,000 cells per well) in triplicates and cultured under standard conditions. At days 1, 3, 6, 9, 12, and 15, the cells were trypsinized and counted in a hemocytometer. The proliferation ability of the cells from each donor was quantified as the area under the curve (AUC) and expressed as arbitrary units (AU). In addition, we calculated population doubling time (PDT) in hours between days 1 and 6 using the following formula PDT = 120 hours×log(2)/(log[N_cellsday6_/N_cellsday1_]).

## 3 CELL DIFFERENTIATION

### 3.1 Osteoblastic differentiation

Human BM‐MSCs at first passage were seeded in a 4‐well plate at a density of 20,000 cells/cm^2^. At 90% confluence, media was replaced with osteoblastic induction media including: 10% FBS, 1% P/S, 5 mM β‐glycerophosphate, 10 nM dexamethasone, 50 μg/mL vitamin C, and 10 nM vitamin D_3_. Osteoblastic induction media were replaced every 2–3 days.

#### 3.1.1 Alizarin red staining for formation of mineralized matrix

After 14 days, the osteoblastic differentiation was assessed by the ability of the cells to form mineralized matrix visualized by alizarin red staining. Briefly, the cells were washed with PBS and fixed with 70% ice‐cold ethanol in −20°C for 1 hour. Subsequently, hBM‐MSCs were washed with H_2_O and incubated with alizarin red (pH = 4.2) for 10 minutes with rotation in room temperature (RT). After that, the cells were washed with PBS for several minutes to remove nonspecific binding. Alizarin red staining was quantified as red intensity using ImageJ software and expressed as AU.

#### 3.1.2 Alkaline phosphatase activity

The cells were washed, fixed with 3.7% formaldehyde‐90% ethanol, and incubated with p‐nitrophenyl phosphate (1 mg/mL) in 50 mM NaHCO_3_ and 1 mM MgCl_2_, pH 9.6 for 20 minutes at 37°C. The reaction was stopped by adding 3 M NaOH. Absorbance was measured at 405 nm and corrected per cell number. The cell number was determined by CellTiter‐Blue reagent (cell viability reagent) for 1 hour at 37°C. The fluorescent intensity of the reagent was measured in FLUOstar Omega plate reader (560ex/590em).

### 3.2 Adipocytic differentiation

Human BM‐MSCs at the first passage were plated at a density of 30,000 cells/cm^2^ in a 4‐well plate for 24 hours. At near full confluence, the media were replaced with adipocytic induction media containing DMEM supplemented with 10% FBS, 1% P/S, 5% horse serum, 1 μM BRL 49653, 3 μg/mL insulin, 100 nM dexamethasone, and 225 μM IBMX. Media were changed every 2–3 days for 2 weeks.

#### 3.2.1 Oil Red O staining of mature AD

Adipocyte differentiation efficiency was determined by lipid droplets area as visualized by Oil Red O staining. The cells were fixed with 4% paraformaldehyde (PFA) for 10 minutes at RT, washed with 3% isopropanol, and incubated with filtrated Oil Red O solution (25 mg of Oil Red O in 5 ml of 100% isopropanol and 3.35 mL H_2_O). Images of the differentiated cells were captured using Olympus optical microscope (×10 magnification objective) and quantified by lipid droplets area (average of six images per sample) using ImageJ software and expressed as AU.

The group of hBM‐MSC populations classified as OB had osteoblastic differentiation values higher than median and adipocytic differentiation values lower than median. For the AD group, adipocytic differentiation was higher than median values and osteoblastic differentiation values were lower than median. All values were based on median values of quantified alizarin red intensity staining (osteoblastic differentiation) and percentage of lipid droplets area (adipocytic differentiation).

### 3.3 Flow cytometry

Human BM‐MSCs were trypsinized and washed with PBS (without Ca^2+^ and Mg^2+^) with 2% FBS. The cells were incubated with primary fluorophore‐conjugated antibodies as follows: CD44‐PE, CD73‐PE, CD90‐PE, CD105‐PE, CD146‐PE, CD271‐FITC, ALPL‐APC, for 25 minutes in 4°C. After the incubation, cells were washed twice to remove unspecific antibody binding and analyzed using BD LSR II Flow Cytometer with BD FACSDiva software and data were analyzed with Kaluza Flow Cytometry Analysis Software Version 1.3 (Beckman Coulter).

### 3.4 Cell morphology analysis

#### 3.4.1 Immunocytochemistry

The cells were trypsinized and 1,000 cells/well were seeded in a 96‐well black plate (CellCarrier‐96, PerkinElmer) in S‐MEM media. After 48 hours, the cells were washed with PBS and fixed with 4% PFA for 10 minutes at RT. The cells were permeabilized with buffer (0.3 M Tris‐glycine and 0.25% Triton‐X in PBS) for 15 minutes (RT), incubated in blocking buffer (1% bovine serum albumin, 0.5% goat serum in PBS) for 1 hour at RT, followed by incubation with antibody for α‐tubulin (dilution 1:500). The cells were washed and incubated with AlexaFluor 488 (dilution 1:1000) for 1 hour followed by incubation with Phalloidin (35 μM for 2 hours at RT). Cell nuclei were visualized using DAPI.

#### 3.4.2 Image analysis

Images of the hBM‐MSCs cultured from each individual donor (15 different areas per well in 9 wells) were acquired using the Operetta high‐content screening system (PerkinElmer, objective ×10 magnification, N.A 0.3). Cell and nucleus morphology parameters including area, roundness, width, length, and ratio of width to length were analyzed using Harmony 3.1 software by employing a predefined protocol based on the building blocks method, which includes nuclear and cellular segmentation, as shown in Figure S1. In addition to cell morphology features, texture parameters of the cytoskeletal fibers—SER analysis including nine patterns (**S**pot, **E**dge, **R**idge, Saddle, Valley Hole, Bright, Dark) at 1px scale were determined to measure the differences in cytoskeletal and nuclear structure. Each morphological parameter was quantified using an average of at least 1,000 cells per donor sample. The list and the description of all measured and quantified cell morphology and texture features are defined in Table S1.

### 3.5 Reagents

Lymphoprep (StemCell Technologies, 1114545), Minimum Essential Media (MEM, Gibco, 31095‐029), Dulbelcco's Modified Eagle Medium (DMEM, Gibco, 31966), FBS (ThermoFisher, 10270106, lot:42F0266K), GlutaMAX (Gibco, 35050‐038), Non‐Essential Amino Acids (MEM NEAA, Gibco, 11140‐035), Trypsin–EDTA (Invitrogen, 25300062), β‐glycerophosphate (Calbiochem, 35675), Dexamethasone (Sigma, D4902), Vitamin C (Wako, 013‐12061), Vitamin D_3_ (kind gift from Leo Pharma), p‐nitrophenyl phosphate (Sigma, 71768), Alizarin Red (Sigma, A5533), Oil Red O (Sigma, O0625), horse serum (Sigma, H1270), rosiglitazone (BRL, Cayman Chemical, 71740), insulin (Sigma, I9278), 3‐isobutyl‐1‐methylxanthine (IBMX, Sigma, I5879), anti‐CD44 (Beckman Coulter, A32537), anti‐CD73 (BD Bioscience, 550257), anti‐CD90 (Beckman Coulter, IM3600U), anti‐CD105 (Beckman Coulter, A07414), anti‐CD146 (Beckman Coulter, A07483), anti‐CD271 (BioLegend, 345104), alkaline phosphatase antibody (ALPL, R&D Systems, FAB1448A), CellTiter‐Blue cells viability assay reagent (Promega, G8081), Phalloidin (Sigma, P1951), mouse anti‐human α‐tubulin (Sigma, T‐9026), DAPI (ThermoFisher, 62248), Alexa Fluor 488 (ThermoFisher, A11001).

### 3.6 Data analysis

#### 3.6.1 Bivariant analysis

Statistical analyses of the correlation between variables were performed using the Spearman two‐tailed correlation test (*r*_*s*_ = Spearman correlation coefficient). For the correlation analysis, outliers were identified and removed using the ROUT method, which detects outliers from nonlinear regression, based on the maximum false discovery rate *Q* = 1%. The number of independent donors (n) in each correlation analysis is described in the Results section and in each figure. Differences between groups were tested by unpaired two‐tailed Student's *t*‐test. All analyses were performed using GraphPad Prism 7.1 software. Statistical significance was considered when *P* < .05.

#### 3.6.2 Multivariable analysis

A stable multivariable linear regression model was created for predicting the osteogenic and log transformed adipogenic differentiation outcome and performed for the whole data set of 56 donors. Due to the large number of potential morphological predictors, we applied the variable selection procedure introduced by Meinshausen.^34^ This procedure allows to control the expected number (PFER) of selected variables that represent uninformative predictors, that is, one controls the number of false discoveries. The central building block of this procedure is a regression modeling approach that allows optimal selection of a predetermined number, *q*, of explanatory variables. In this case, the penalized regression “Lasso” method^35^ was used, that generated 50 random subsamples of the actual data and fitted to each subsample a regression model and thus obtained 50 sets of *q* predictor variables. Based on these sets, we estimated the selection probability of the predictor variables via their relative frequency of having been chosen. Finally, we retained only the stable predictors, with selection probabilities larger than a prechosen threshold probability θ. The chosen *q* and θ determined an upper limit for the PFER. We chose PFER = 2 and θ = 0.75 and determined q consistent with the PFER. The choice of θ was shown to be uncritical. Furthermore, we calculated Akaike's Information Criteria (AIC), which denotes the predictive power of the model employing new data set. For determination of the individual prediction value of the variables, the estimated AUC for the receiver operator characteristic was calculated.

## 4 RESULTS

### 4.1 Cultured hBM‐MSCs exhibit heterogenous cell and nucleus morphology

Our initial analysis of cell morphology (illustrated in Figure 1) demonstrated that cultured hBM‐MSCs exhibited intra‐ and inter individual heterogeneity in cell and nucleus morphology. Photomicrographs illustrate examples of variations in cell morphology (Figure 1A) and nuclear morphology (Figure 1B) in cells derived from two individual donors. Figure 1A (left) shows cells of donor #1, that were generally smaller compared with cells of donor #2 (Figure 1A right). Intra‐ and inter donor variations of cell size can be appreciated from the frequency distribution of cell area of the entire cell population (Figure 1C). Similarly, Figure 1B illustrates variations in nucleus morphology with two contrasting nuclear geometries: oval (left) versus rounded‐shaped nuclei (right). Quantitative analysis of individual nuclear shape expressed as nucleus width to length ratio is illustrated in the frequency distribution of the whole cell population (Figure 1D). For the whole cohort examined (n = 56 donors), cultured hBM‐MSCs exhibited large inter donor variability as shown in the frequency distribution of the mean values of cell areas (Figure 1E) and nucleus width to length ratio (Figure 1F). Thus, the presence of inter‐individual morphological heterogeneity of cultured hBM‐MSCs allowed us to examine the relationship between cell and nucleus morphology and cellular functions.

### 4.2 CD marker expression correlate with cellular and nuclear morphology

**Figure 2:**
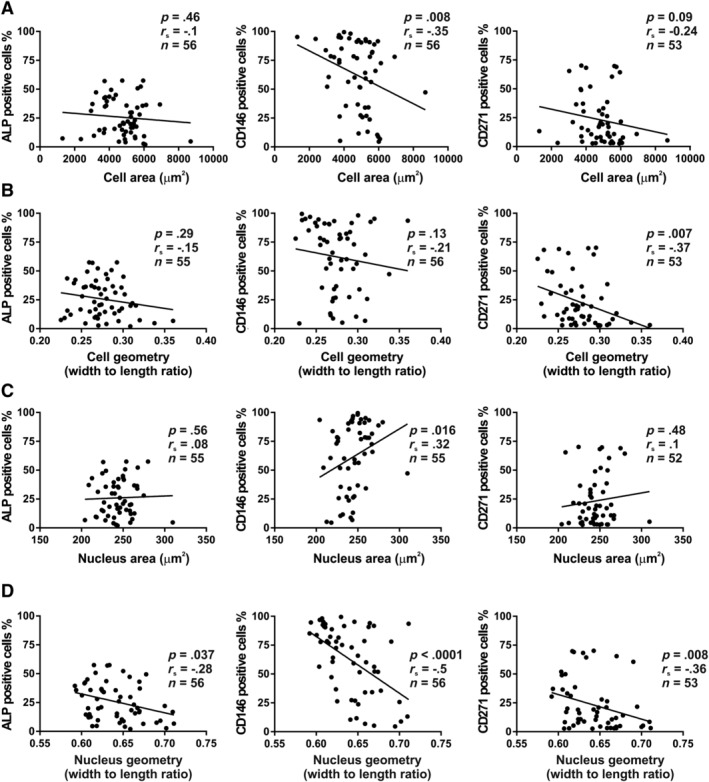
Relationship between expression of membrane markers of hBM‐MSCs and cell and nucleus morphology. A, Correlation between mean value of cell areas of hBM‐MSCs population from each donor and number of ALP+, CD146+, or CD271+ cells. B, Correlation between mean value of hBM‐MSC population ratio of cell width to length from each donor and number of ALP+, CD146+, or CD271+ cells. C, Correlation between mean value of hBM‐MSC population nucleus areas from each donor and number of ALP+, CD146+, or CD271+ cells. D, Correlation between mean value of hBM‐MSC population ratio of nucleus width to length, from each donor and the number of ALP+, CD146+, or CD271+ cells. Each dot represents the average value of cultured cells derived from an individual donor and n indicates the number of donors tested. ALP, alkaline phosphatase; hBM‐MSCs, Human bone marrow‐derived stromal (mesenchymal) stem cells

Human BM‐MSCs express a number of CD surface markers, some of which suggested by International Society for Cellular Therapy (ISCT).^5^, ^36^ We analyzed the expression of CD44, CD73, CD90, and CD105 markers on the initial samples of 15 donors, to ensure that the isolated cells fulfill the minimal criteria for hBM‐MSCs. We found that CD44, CD73, CD90, and CD105 were uniformly expressed, in the whole cell populations with minimal inter individual variation (mean ± SD): CD44: 99.87 ± 0.21%; CD73: 99.75 ± 0.28%; CD90: 99.24 ± 0.81%; CD105: 99.88 ± 0.08%. In contrast, we identified three markers of cultured hBM‐MSCs: ALP, CD146 (melanoma cell adhesion molecule, MCAM), and CD271 (low‐affinity nerve growth factor receptor alpha)^37^, ^38^, ^39^ that exhibited variable expression among donors (mean and SD 26 ± 16%; 62 ± 30%; 26 ± 25%, respectively), and therefore allowed testing of their influence on variations in cell morphology. Among the three markers, we observed significant negative correlation between number of CD146+ cells and cell area (*r*_s_ = −0.35, *P* < .01, n = 56) (Figure 2A). CD271 and ALP showed a nonsignificant tendency of negative correlation with cell area (Figure 2A). On the other hand, percentage of CD271+ cells were negatively correlated with cell shape (width to length ratio) (*r*_s_ = −0.37, *P* < .01, n = 53) (Figure 2B). Moreover, percentage of CD146+ cells were positively correlated with nuclear area (*r*_s_ = 0.32, *P* < .05, n = 55) (Figure 2C). Interestingly, we observed that the hBM‐MSCs that are ALP+, CD146+, and CD271+ exhibited significant negative correlations with nucleus width to length ratio: ALP+ (*r*_s_ = −0.28, *P* < .05, n = 56), CD146+ (*r*_s_ = −0.5, *P* < .0001, n = 56), and CD271+ (*r*_s_ = −0.36, *P* < .01, n = 53; Figure 2D).

### 4.3 Relationship of cell morphology and biological functions of hBM‐MSCs

**Figure 3:**
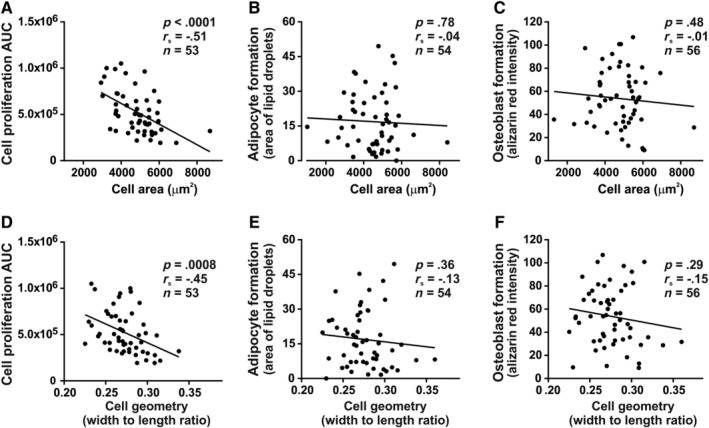
Relationship between cell morphology and functionality of cultured hBM‐MSCs. Cell area estimated from α‐tubulin staining of the cells. Cell size was correlated with (A) proliferative capacity of the cells but not with (B) mature adipocyte formation visualized by Oil Red O staining of intracellular lipids, or (C) mature osteoblast formation evidenced by alizarin red staining of mineralized matrix. Cell geometry expressed as the ratio of width to length exhibited significant correlation with cell proliferation (D) but not with (E) adipocytic differentiation or (F) osteoblastic differentiation in vitro. Each dot represents the average value of cultured cells from a single donor and n indicates the number of donors tested. Variables displayed on y‐axis are expressed in arbitrary units (AU). The proliferation was quantified as area under the curve (AUC). hBM‐MSCs, Human bone marrow‐derived stromal (mesenchymal) stem cells

We examined the correlation between morphology of hBM‐MSCs (based on α‐tubulin staining, at baseline undifferentiated state) and cellular functions. Mean cell area (Figure 3A‐C) and cell shape (cell width/length) (Figure 3D‐F) of cell populations, exhibited significantly negative correlation with the cell proliferation capacity expressed as AUC of short‐term proliferation (*r*_s_ = −0.51, *P* < .0001, n = 53 and *r*_s_ = −0.45, *P* = .0008, n = 53, respectively). The AUC of cell proliferation curves was generally used as a good metric to summarize the growth curve.^40^ Nevertheless, we also calculated the average value of PDT for all donor cells (76.5 ± 34 hours), which was similar to previously reported PDT for primary hBM‐MSCs (between 72 and 120 hours).^41^, ^42^, ^43^ Furthermore, we found a strong negative correlation between AUC and PDT values for analyzed cell populations (*r*_s_ = −0.73, *P* < .0001, n = 53, Figure S2A). Consistent with the AUC data, we observed a positive correlation with PDT and mean cell area (*r*_s_ = 0.56, *P* < .0001, n = 53) and cell geometry (*r*_s_ = 0.32, *P* = .02, n = 53) which can be seen in Figure S2B‐C.

In contrast to cell proliferation findings, adipocyte differentiation capacity (estimated by the lipid droplets area of mature AD) or OB differentiation (based on mineralized matrix formation) did not correlate with cell area (*P* = .78 and *P* = .48, respectively, Figure 3B, C) or cell shape (*P* = .36 and *P* = .29, Figure 3E, F). Similar findings were obtained when cell morphology was determined from F‐actin staining (Figure S3).

### 4.4 Relationship of cytoskeletal texture and biological functions of hBM‐MSCs

**Figure 4:**
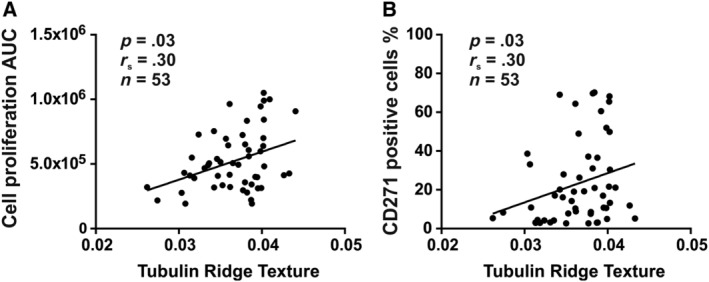
Relationship between the tubulin texture and hBM‐MSC functions. The cytoskeletal fiber texture was determined following tubulin staining. The ridge pattern of cytoskeletal texture exhibited significant positive correlation with the proliferation activity (A) and the percentage of CD271+ cells (B). Each dot represents the average value of cultured cells derived from a single donor and n indicates the number of donors tested. The proliferation was quantified as area under the curve (AUC). hBM‐MSCs, Human bone marrow‐derived stromal (mesenchymal) stem cells

High‐content image analysis provides information regarding cellular texture parameters that reflect the cytoskeletal status of the cells. Analysis of the cytoskeletal fiber texture demonstrated that the ridge pattern of tubulin staining was positively correlated with proliferation capacity of hBM‐MSCs (*r*_s_ = 0.30, *P* < .05, n = 53) (Figure 4A). In addition, we observed that the tubulin ridge texture was positively correlated with percentage of CD271+ cells (*r*_s_ = 0.30, *P* < .05, n = 53) (Figure 4B). We did not observe any significant correlations between cytoskeletal texture and osteoblastic or adipogenic differentiation capacity of hBM‐MSCs or with the percentage of CD146+ or ALP+ cells (data not shown).

### 4.5 Relationship between nucleus morphology and biological functions of hBM‐MSCs

**Figure 5:**
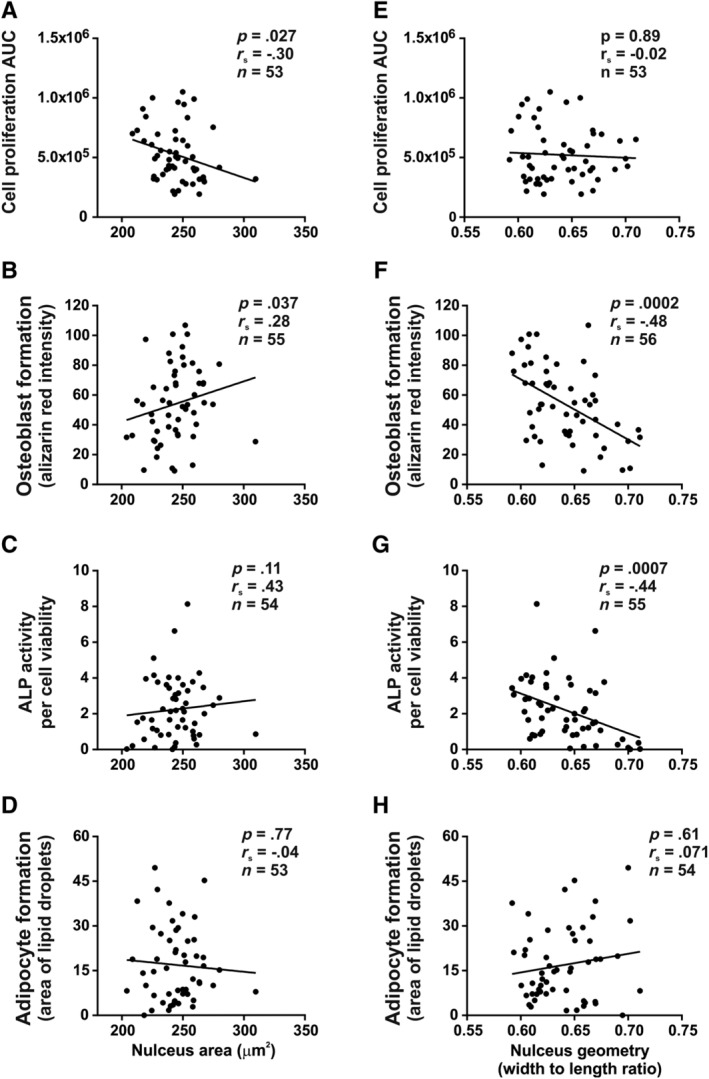
Relationship between nucleus morphology and hBM‐MSC functions. Nucleus area was significantly correlated with (A) proliferation ability of hBM‐MSCs and (B) mature osteoblast formation evidenced by alizarin red staining of mineralized matrix. C, ALP activity following osteoblastic differentiation induction or (D) adipocytic differentiation potential did not exhibit significant correlation with nucleus size. Nucleus geometry (ratio of nucleus width to length) showed a significant negative correlation with mature osteoblast formation (F) and with ALP activity (G). No significant correlation was observed between nucleus geometry and other cell functions including (E) proliferation and (H) mature adipocyte formation. ALP, alkaline phosphatase; hBM‐MSCs, Human bone marrow‐derived stromal (mesenchymal) stem cells

We further examined the relationship between nucleus morphology and functions of cultured hBM‐MSCs. Nucleus area exhibited significant negative correlation with cell proliferation capacity (Figure 5A) (*r*_*s*_ = −0.30, *P* = .027, n = 53) and positive correlation with osteoblastic differentiation potency (*r*_*s*_ = 0.28, *P* = .037, n = 55) (Figure 5B) suggesting that cultures enriched in cells with smaller nuclear area exhibited higher cell proliferation, whereas cells with larger nuclei were more prone to osteoblast differentiation. We did not detect significant correlation between nucleus area and ALP activity (Figure 5C) or between nucleus area and adipocyte differentiation (Figure 5D). Interestingly, nucleus shape (width to length ratio) was negatively correlated with osteoblast differentiation and demonstrated that cultures enriched in cells with oval‐shaped nuclei exhibited enhanced osteoblastic differentiation (*r*_*s*_ = −0.48, *P* < .001, n = 56) (Figure 5F). Furthermore, a negative correlation between ALP activity and nuclear geometry was observed (*r*_*s*_ = −0.44, *P* < .001, n = 55) (Figure 5G). We did not detect significant correlation between nucleus shape and cell proliferation or adipocyte differentiation (Figure 5E, H).

**Figure 6:**
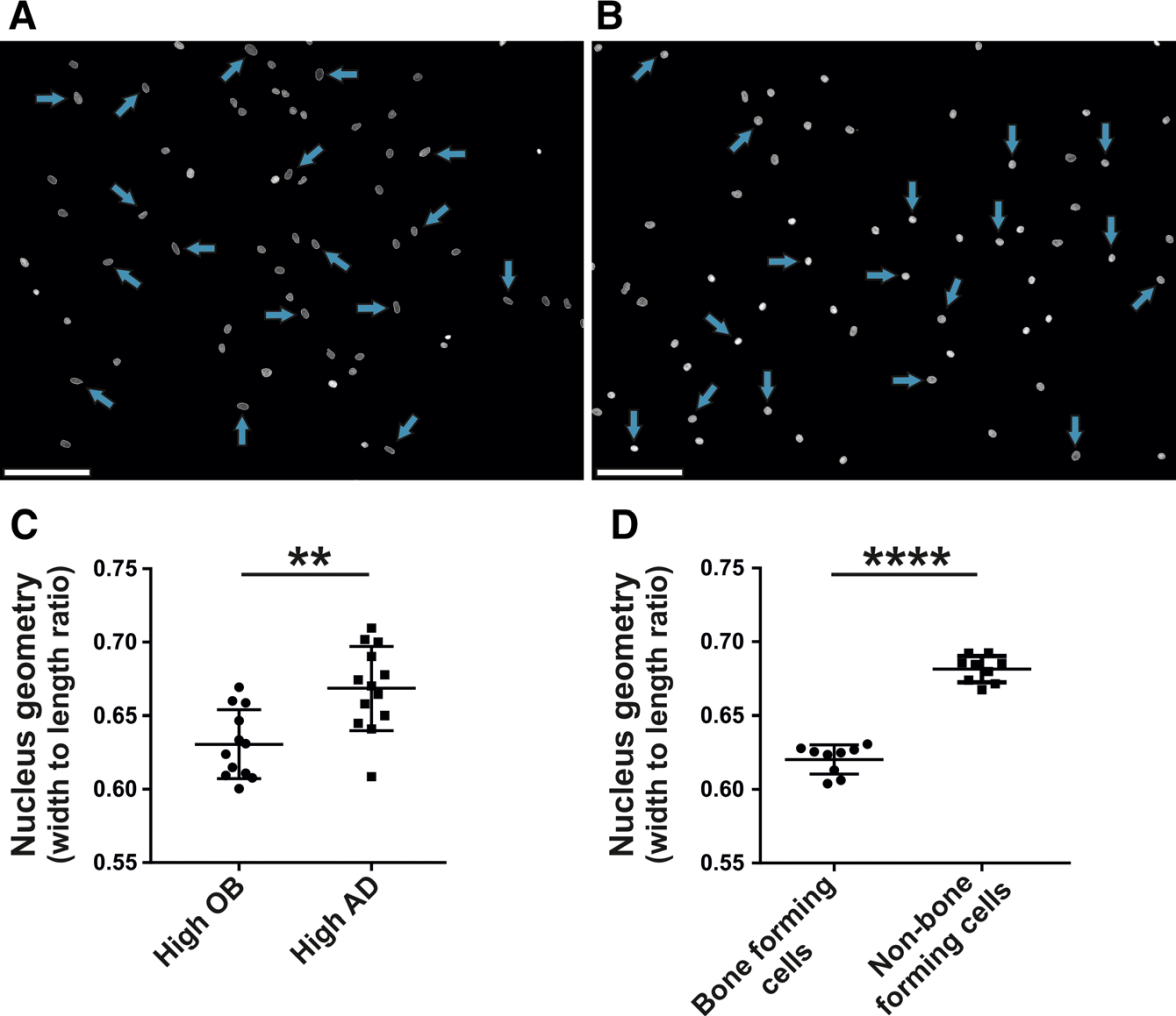
Relationship between nucleus geometry and hBM‐MSC functions. Photomicrographs illustrating nuclear geometry of (A) cultured hBM‐MSCs with enhanced osteoblastic differentiation and decreased adipocyte differentiation (high OB) and (B) a population with enhanced adipocytic differentiation and decreased osteoblast differentiation (high AD). Arrows show examples of round nuclei (high width to length ratio) or oval nuclei (low width to length ratio). C, Cultures of hBM‐MSCs established from independent donors were grouped based on their differentiation capacities as either high OB or high AD and compared with respect to mean nucleus geometry. D, The nucleus geometry of two clonal populations of hBM‐MSCs with either high or low in vivo bone formation potential.^9^ Nuclei were stained with DAPI. Data are shown as mean ± SD with **P < .01; ****P < .0001. hBM‐MSCs, Human bone marrow‐derived stromal (mesenchymal) stem cells

To further analyze the importance of nucleus geometry, we classified all donors based on the ability of their cells to differentiate into either OB or AD. We compared population mean values of nucleus shape between the two groups with contrasting differentiation potential: high OB group (high OB, low AD) and high AD group (high AD, low OB). The photomicrographs in Figure 6 illustrate nucleus shape of representative populations of hBM‐MSCs classified as high OB (Figure 6A) or high AD (Figure 6B) groups.

As illustrated in Figure 6C, OB differentiation is enhanced in cultures enriched in cells with oval‐shaped nuclei, whereas enhanced AD differentiation was observed in cultures enriched in more round‐shaped nuclei. In addition, we calculated the value of variable prediction as determined by the estimated AUC = 0.84; 0.68 to 1.00 (97.5% confidence interval). This suggests that the predictive power is modest on average but significantly higher than expected by random selection (AUC = 0.5 for random data). To corroborate the relevance of our findings, we examined changes in nuclear morphology in two clonal hBM‐MSC populations previously defined in our laboratory as exhibiting enhanced or reduced capacity for in vivo bone formation.^9^ As seen in Figure 6D, hBM‐MSCs with high in vivo bone forming capacity were enriched in oval‐shaped nuclei.

### 4.6 Multivariable analysis to identify predictors of hBM‐MSC differentiation potential

**Figure 7:**
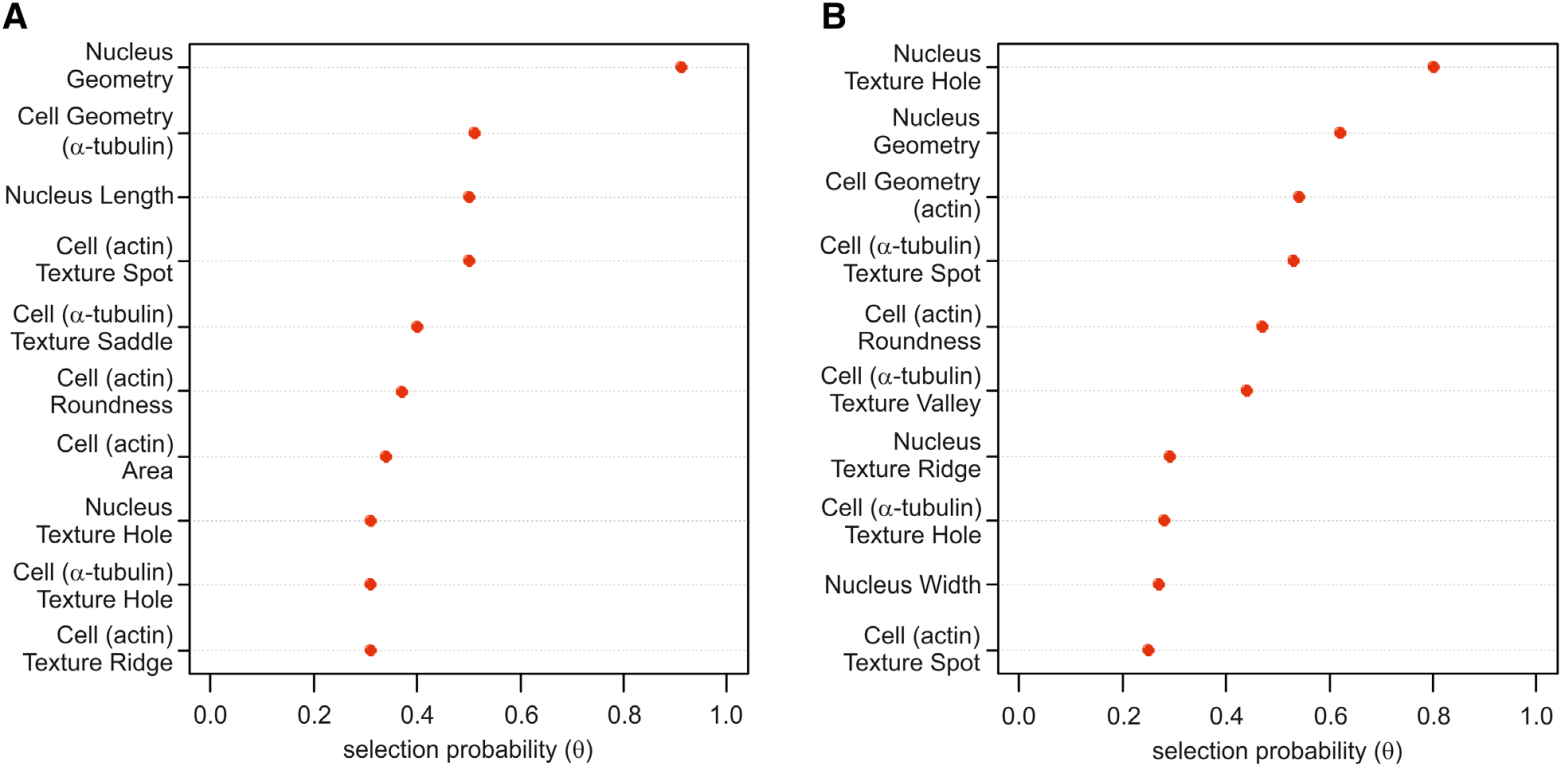
Multivariable analysis (stability selection model) of the morphological features for cultured hBM‐MSCs that are predictive for osteogenic or adipogenic differentiation. The y‐axis lists the parameters that have been chosen by the model as the most stable predictors for (A) osteoblastic or (B) adipocytic differentiation. The x‐axis demonstrates the selection probability of the chosen variables with θ as a threshold probability. hBM‐MSCs, Human bone marrow‐derived stromal (mesenchymal) stem cells

To identify the morphological predictors for hBM‐MSC differentiation potential, we performed a multivariable analysis that included all measured morphological and texture features of cells and nuclei. Using a stability selection procedure, we identified parameters with explanatory power of the primary outcome of osteoblastic (Figure 7A) and adipocytic (Figure 7B) differentiation. We found that nucleus geometry is the morphological feature that is highly stable predictor for OB differentiation potential of the cells (θ > 0.75) and negatively associated with osteoblast differentiation.

For adipocyte differentiation, the nuclear “Hole” texture pattern was selected as a highly predictive variable (θ > 0.75) with negative influence on adipocytic differentiation potential. However, “Hole” texture pattern, did not reach statistical significance in bivariant analysis, (Figure S4). Moreover, two cell morphology features were identified as variables of moderate explanatory power (θ > 0.5) associated with AD formation namely nucleus width to length ratio (positively associated) and cell width to length ratio (negatively associated).

The relative performance of the model was measured by AIC that determines the predictive power of the model when applying it to a new data set. We performed the analysis comparing various models (m0, m1, m2) for osteogenic and adipogenic outcomes including different combination of covariates. For osteogenic outcome, models were as follow: m0 (intercept only, prediction only based on the mean value), nucleus geometry (m1) and combination of identified top four valuables including nucleus geometry, cell geometry (α‐tubulin), nucleus length, and cell texture Spot (m2). Similarly, for adipogenic outcome, we included: m0 (intercept only), nucleus texture Hole (m1), and top four identified variables: nucleus texture Hole, nucleus geometry, cell geometry (actin), and cell texture Spot (m2). Our data demonstrated (Table S2) that by adding the highly predictive variable (θ > 0.75) identified by the multivariable analysis, the prediction (m1) of both osteogenic and adipogenic differentiation outcomes was improved. For osteogenic outcome, including additional variables did not improve the prediction (m2), in contrast to the adipogenic outcome, where including the top four variables gave the best prediction model (m2).

## 5 DISCUSSION

Cultured hBM‐MSCs represent a heterogenous population of cells with respect to their in vivo differentiation potential which may lead to inconsistent and variable results when being used in clinical trials.^5^ The aim of this study was to identify cellular morphological characteristics as “cell quality markers” that are predictive for proliferation and differentiation capacity of hBM‐MSC populations and can be used to choose suitable cells for clinical transplantation protocols. Using high‐content image analysis of cultured hBM‐MSCs, at a single‐cell resolution, we identified a number of parameters that are indicative of their differentiation potential. Among these, nucleus morphology is a strong predictor of cell differentiation capacity where cells possessing oval‐shaped nuclei poised for osteoblastic differentiation and cells with more round‐shaped nuclei are poised for adipocytic differentiation.

We observed as expected, that there exist large intra‐ and inter‐individual variations in the morphological characteristics of cultured hBM‐MSCs. The inter individual variations allowed us to identify predictive factors that are associated with biological functions relevant for their use the clinic. Similar to our findings, a number of studies of cultured hBM‐MSCs reported the presence of inter individual cellular heterogeneity with respect to their differentiation capacity that was correlated with donors characteristics^11^, ^44^, ^45^ as well as intrinsic variations within the cultured cells that may reflect functional heterogeneity of the cultured cells.^37^, ^46^, ^47^, ^48^ Our study extends these results and provides a systemic approach of examining the relationship between morphological characteristics observed at a single‐cell resolution and differentiation capacity of the cells in a large cohort of donors.

Canonical hBM‐MSCs are defined by expression of a number of cell surface markers^14^ including the standard CD markers recommended by ISCT. In our initial screening, we observed that these markers are homogenously expressed by the majority of the cells in all donors examined and therefore not suitable as predictors for determining the functional variations among donors. In contrast, more specific hBM‐MSC markers, for example, CD146, ALP, and CD271,^37^, ^38^, ^49^ exhibited heterogenous expression among donors, which allowed testing their predictive value with respect to differentiation ability of the cells. We observed that cells that are positive for these markers exhibited elongated‐shaped of nuclei and enriched in osteoblastic lineage committed cells. Our data suggest that isolation a subset of cultured hBM‐MSCs, based on these markers, may be useful for obtaining cells with high osteoblast differentiation capacity for clinical trials of bone regeneration. In addition, hBM‐MSC populations with increased number of CD146‐positive cells exhibited smaller cell size. Colter et al^21^ also observed differences in cell size of cultured hBM‐MSCs and found that small rounded cells exhibited a stemness phenotype as shown by in vitro differentiation capacity and self‐renewal. Thus, previous work also show that cell size and nucleus geometry can be applied to identify hBM‐MSCs with self‐renewal characteristics.

Cell proliferation capacity is an important prerequisite for regenerative therapy protocols as large numbers of cells are needed. Also, highly proliferative hBM‐MSCs exhibit better engraftment in preclinical mice models.^50^ The hBM‐MSCs in the current study had a similar PDT (76.5 ± 34 hours) as observed in previous studies.^41^, ^42^, ^43^ We further observed that hBM‐MSC cultures enriched in small cell and nuclei exhibited greater proliferative capacity. Our data support previous findings reported by Merklein et al^32^ that a positive correlation exists between cell area of primary hBM‐MSCs and time to reach 50% confluence, indicating the cells with smaller area exhibit higher proliferation rate.^32^ Previous studies have shown, hBM‐MSCs obtained from older donors are generally larger in size and exhibit reduced proliferative capacity and in vitro senescence during long‐term cultures.^13^, ^51^ Also, Colter et al reported that the subpopulation of small hBM‐MSCs exhibited significantly enhanced proliferation rate compared with subpopulations with larger cells.^21^ The relationship between small cell size and proliferative potential has also been reported in other cell types.^52^, ^53^ It is plausible that cell size and nuclear size can be used as surrogate measures of replicative potential of cultures.

Osteoblast differentiation is the default differentiation pathway of cultured hBM‐MSCs^54^ and is the basis for their use in treatment of bone injuries. An inverse relationship has been observed between hBM‐MSC capacity for differentiation into osteoblastic versus adipocytic cells.^55^ In our study, the capacity for differentiation into AD was considered an “undesired” outcome of hBM‐MSC differentiation. Interestingly, in multivariate analysis, nuclear geometry was the variable with the most explanatory power for variations in differentiation potential of cultured hBM‐MSCs toward osteoblastic versus adipocytic cells.

Several previous studies have correlated cell morphology of the whole cultured cell population with the progression of stem cell differentiation^23^, ^29^, ^32^, ^56^ or immunosuppressive capacity.^57^, ^58^ These studies actively modified cell shape by culturing the cells on a number of micropatterned surfaces, initiating differentiation or stimulating with IFNγ, which all induces morphological changes in the hBM‐MSCs. These changes could either be used to alter the differentiation outcome or predict the BM‐MSC biology.^23^, ^29^, ^32^, ^56^, ^57^, ^58^ The novelty of the current study lays in the use of naive hBM‐MSC morphology.

The association of cellular shape and functional outcome may be explained by changes in actin dynamics. We have previously demonstrated that changes in cell shape induced by alternations in the actin cytoskeleton structure determine differentiation outcome of hBM‐MSCs with cellular changes associated with actin de‐polymerization led to enhanced adipocytic differentiation, whereas inhibition of actin de‐polymerization increased osteoblast formation of hBM‐MSCs.^25^, ^26^ Furthermore, one study also shows that stimulation of a immunomodulating pathway can lead to a morphological response in BM‐MSCs.^57^, ^58^ Thus, culturing hBM‐MSCs on biomaterials with specific microstructure^59^, ^60^ or stimulation of certain pathways^57^ could be used to obtained clinically relevant cell population with high OB differentiation potential or immunomodulatory properties suitable for clinical transplantation.

Multivariable analysis identified nucleus geometry as the most stable predictor factor of the differentiation capacity of the cells. The cell nucleus has been proposed to function as a mechano‐sensor,^30^, ^61^ where cytoskeleton and nucleoskeleton linkers transmit extracellular and cytoplasmic forces that alter nuclear shape and thus affecting chromatin organization and transcriptional activity^30^, ^62^ as well as cellular differentiation.^63^ In support of this notion, a recent study demonstrated that forcing hBM‐MSCs to alter nuclear geometry by culturing on a micropatterned surface was associated with enhanced histone 3 acetylation,^64^ a factor that is associated with increased osteoblast differentiation.^65^ In addition, changes in nucleus geometry have been reported in rat MSCs to be associated with increase of osteogenic and decrease of adipogenic gene‐marker expression.^24^

Our study has some limitations. We used in vitro differentiation assay as a surrogate marker for the in vivo bone‐forming capacity. Several markers of in vitro differentiation to OB are not predictive for bone forming capacity of the cells.^9^ However, to corroborate our findings, we demonstrated that in vivo bone forming capacity is positively associated with the presence of elongated nuclei (low width to length ratio) based on data on a hBM‐MSC cell line with known high heterotopic bone forming capacity (Figure 6D). Also, standard culture conditions were used for expansion and differentiation of hBM‐MSCs. Confirmation studies are needed to test clinical grade hBM‐MSCs cultured under GMP conditions.

Routine clinical use of hBM‐MSCs in therapy requires development of easy and noninvasive assays for determining “cell quality.” We have previously demonstrated that Raman spectroscopy can be used to confirm the normal, nontransformed phenotype of hBM‐MSCs prior to clinical transplantation.^66^ In the current study, we demonstrate that using a limited number of morphological characteristics, it is possible to predict the proliferative capacity and the differentiation potential of hBM‐MSCs. The clinical efficacy of using these criteria as quality parameters for transplanted hBM‐MSCs remains to be determined in prospective clinical studies.

## CONFLICT OF INTEREST

H.S. declared consultant/advisory role with Arthrex and ownership interest in Johnson & Johnson. The remaining authors declared no potential conflicts of interest

## AUTHOR CONTRIBUTIONS

J.M.K.: conception and design, collection and assembly of data, data analysis and interpretation, manuscript writing; H.S.: provision of study material; U.H.: data analysis and interpretation; J.B.H.: data analysis and interpretation; M.K.: conception and design, data analysis and interpretation, manuscript writing, final approval of manuscript.

## Supporting information


**Figure S1** Scheme of the experimental design. Human primary bone marrow stromal stem cells (hBM‐ MSCs) were examined for (1) osteoblastic or adipocytic differentiation, cell proliferation, cell membrane marker expression and morphological analysis employing high‐content imaging (Opretta, Perkin Elmer). The cells were stained with DAPI and antibodies for cytoskeletal proteins (actin and tubulin) (2). Images were analyzed based on nuclear and cellular segmentation (3). Border cells were deselected from further analysis (4). Cellular and nuclear features were analyzed (15 areas in 9 wells per sample) and quantified (5) and subsequently correlated with results obtained from functional assays.

**Figure S2** Data showing that PDT correlates with AUC analysis and significantly predicts cell morphology. The values of population doubling time (PDT) **(A)** of BM‐MSCs as expected significantly and negatively correlated with AUC values. PDT of hBM‐MSCs correlated positively with **(B)** cell area and with **(C)** cell geometry.

**Figure S3** Relationship between cell morphology and hBM‐MSC functions. Cell area of hBM‐MSCs based on F‐actin staining is significantly correlated with **(A)** proliferative capacity but not with mature **(B)** adipocyte formation or **(C)** osteoblast formation. Cell geometry expressed as width to length ratio exhibited significant negative correlation with cell proliferation ability, but did not correlate with mature **(E)** adipocyte formation or **(F)** osteoblast formation.

**Figure S4** Relationship between nucleus texture and adipogenic differentiation of hBM‐MSCs. The nucleus texture was determined following DAPI staining. The Hole pattern of nuclear texture exhibited a negative tendency with the adipogenic differentiation potential of BM‐MSCs.

**Table S1**: Supplementary information

**Table S2**: Supplementary information

## Data Availability

The data that support the findings of this study are available on request from the corresponding author. The data are not publicly available due to privacy or ethical restrictions.
