# Supplementary figures and images for: Single‐cell high‐content imaging parameters predict functional phenotype of cultured human bone marrow stromal stem cells
Source: Stem Cells Transl Med. 2019 Nov 23;9(2):189–202. doi: 10.1002/sctm.19-0171 (PMC6988772; doi:10.1002/sctm.19-0171)

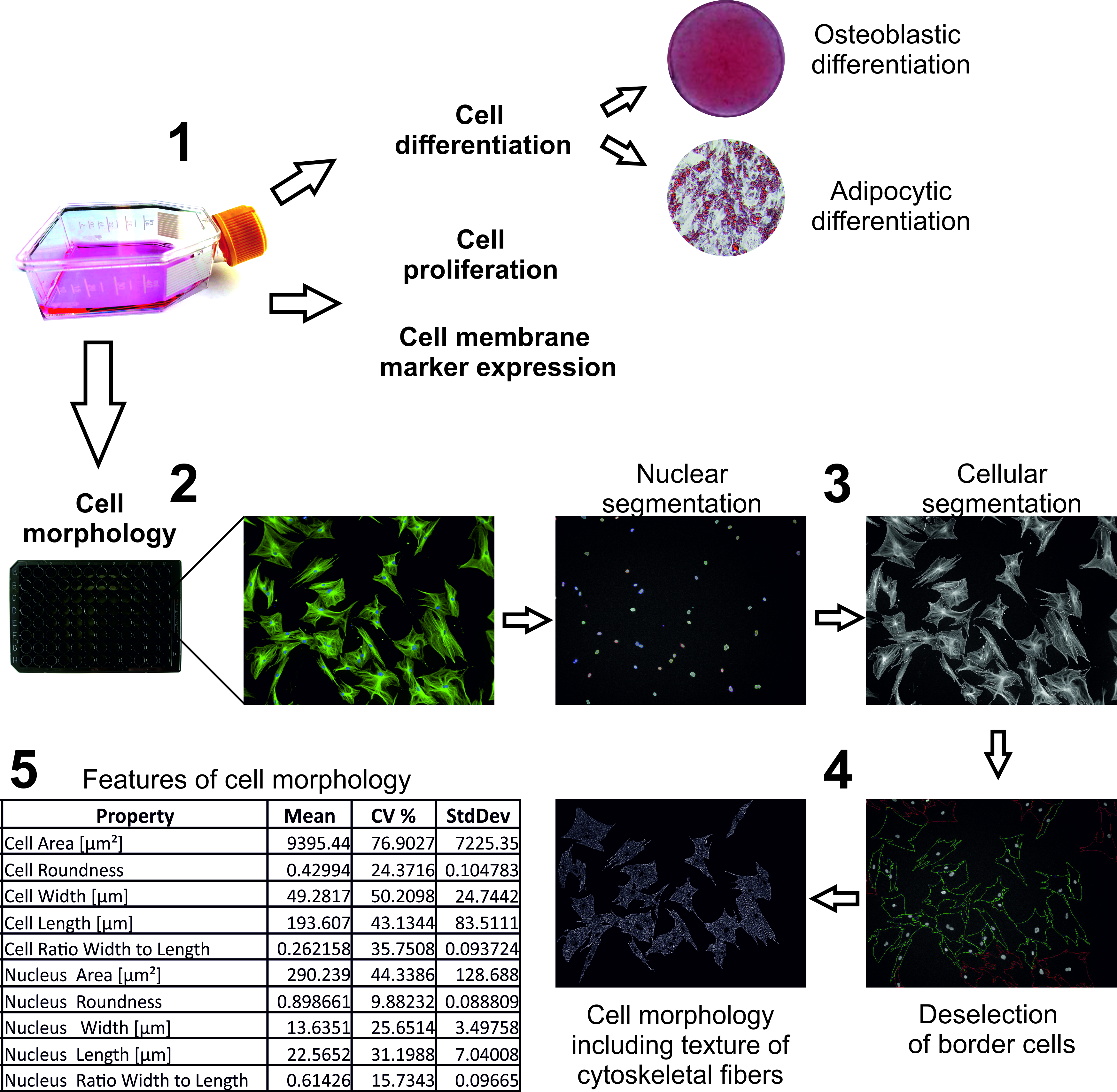

Supplement: Supplementary file 1 — Figure S1 Scheme of the experimental design. Human primary bone marrow stromal stem cells (hBM‐ MSCs) were examined for (1) osteoblastic or adipocytic differentiation, cell proliferation, cell membrane marker expression and morphological analysis employing high‐content imaging (Opretta, Perkin Elmer). The cells were stained with DAPI and antibodies for cytoskeletal proteins (actin and tubulin) (2). Images were analyzed based on nuclear and cellular segmentation (3). Border cells were deselected from further analysis (4). Cellular and nuclear features were analyzed (15 areas in 9 wells per sample) and quantified (5) and subsequently correlated with results obtained from functional assays. [file SCT3-9-189-s001.tif]

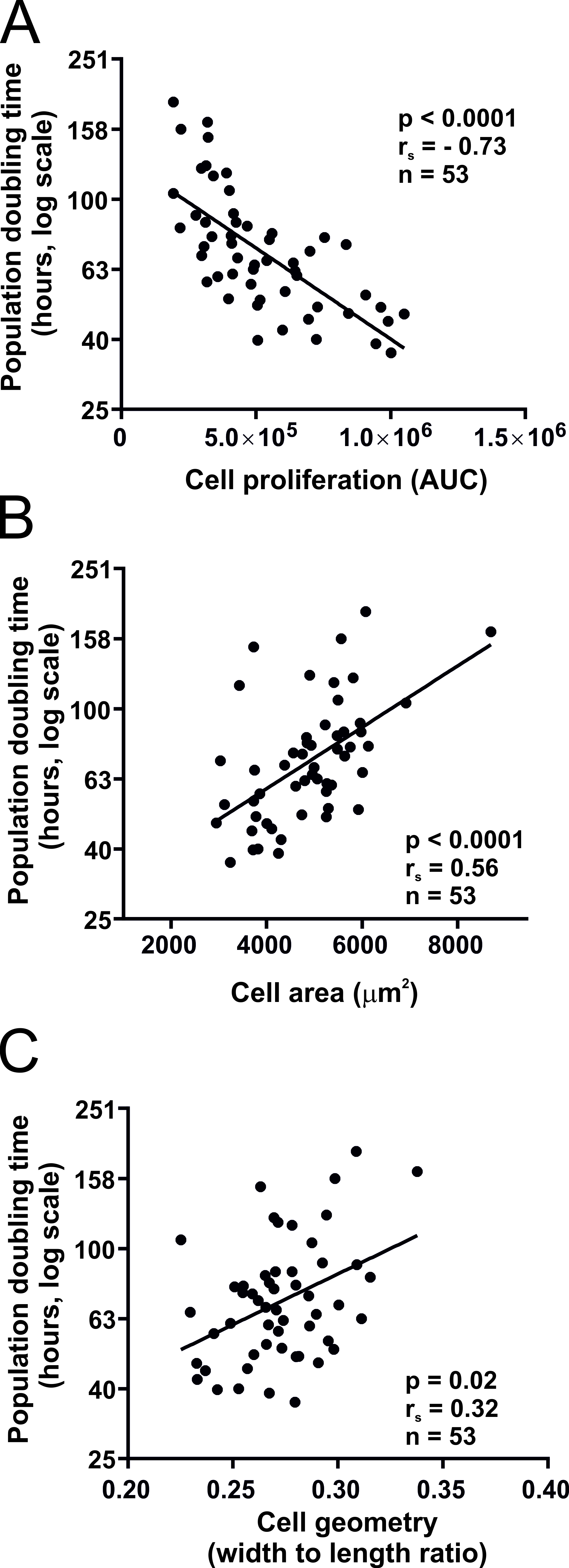

Supplement: Supplementary file 2 — Figure S2 Data showing that PDT correlates with AUC analysis and significantly predicts cell morphology. The values of population doubling time (PDT) (A) of BM‐MSCs as expected significantly and negatively correlated with AUC values. PDT of hBM‐MSCs correlated positively with (B) cell area and with (C) cell geometry. [file SCT3-9-189-s002.tif]

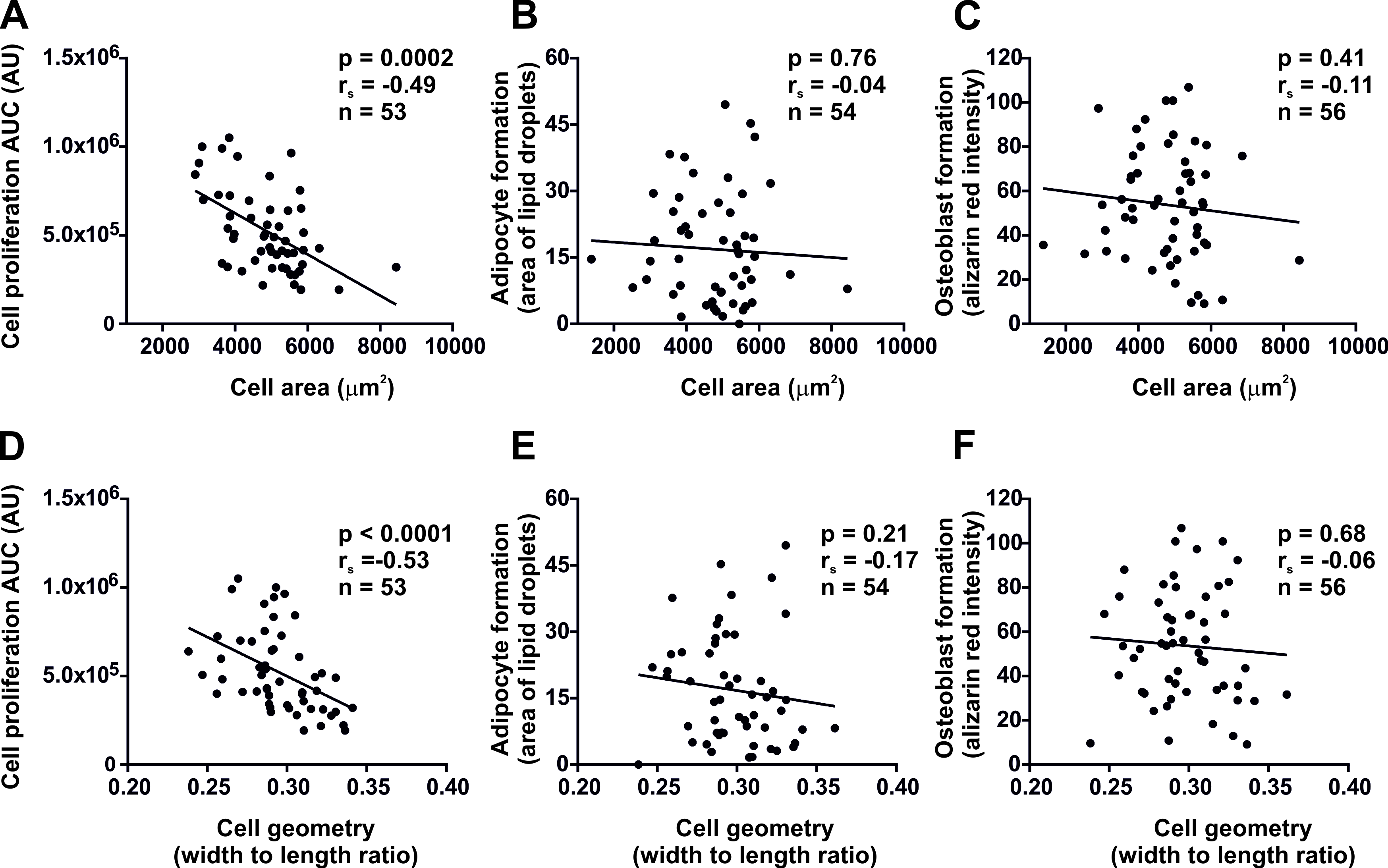

Supplement: Supplementary file 3 — Figure S3 Relationship between cell morphology and hBM‐MSC functions. Cell area of hBM‐MSCs based on F‐actin staining is significantly correlated with (A) proliferative capacity but not with mature (B) adipocyte formation or (C) osteoblast formation. Cell geometry expressed as width to length ratio exhibited significant negative correlation with cell proliferation ability, but did not correlate with mature (E) adipocyte formation or (F) osteoblast formation. [file SCT3-9-189-s003.tif]

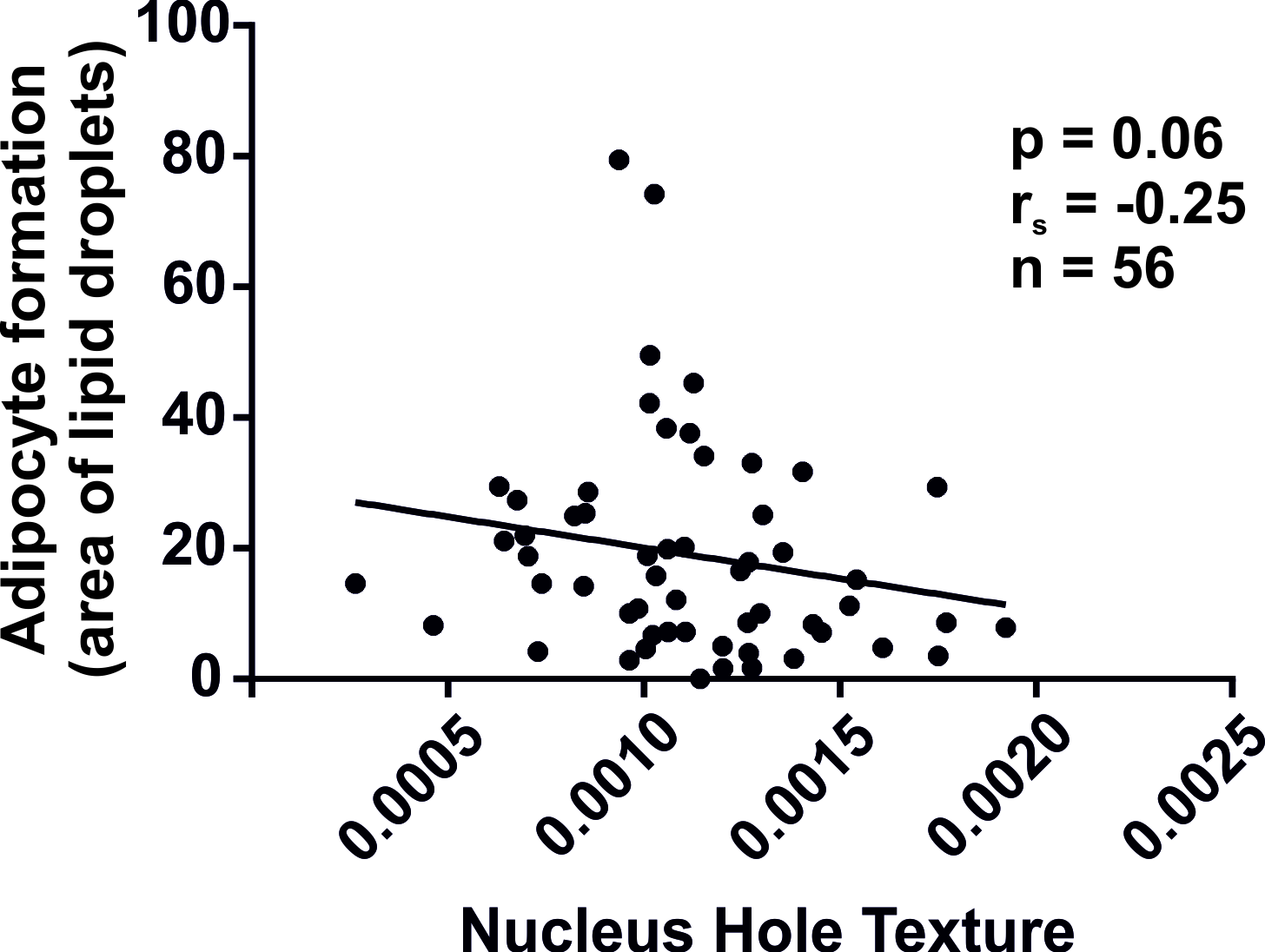

Supplement: Supplementary file 4 — Figure S4 Relationship between nucleus texture and adipogenic differentiation of hBM‐MSCs. The nucleus texture was determined following DAPI staining. The Hole pattern of nuclear texture exhibited a negative tendency with the adipogenic differentiation potential of BM‐MSCs. [file SCT3-9-189-s004.tif]
