# Supplementary material for: Single‐cell high‐content imaging parameters predict functional phenotype of cultured human bone marrow stromal stem cells
Source: Stem Cells Transl Med. 2019 Nov 23;9(2):189–202. doi: 10.1002/sctm.19-0171 (PMC6988772; doi:10.1002/sctm.19-0171)
Supplement: Supplementary file 5 — Table S1: Supplementary information [file SCT3-9-189-s005.docx]

**Supplementary Table 1 List of morphological parameters**

| **Name of parameter** | **Description** | **Type of analysis** | **Staining** |
| --- | --- | --- | --- |
| Nucleus Area | Nucleus area | Nucleus morphology | nucleus - DAPI |
| Nucleus Roundness | Nucleus roundness | Nucleus morphology | nucleus - DAPI |
| Nucleus Width | Nucleus width | Nucleus morphology | nucleus - DAPI |
| Nucleus Length | Nucleus length | Nucleus morphology | nucleus - DAPI |
| Nucleus Geometry | Ratio of nucleus width to length | Nucleus texture | nucleus - DAPI |
| Nucleus Spot | Spot pattern of nucleus staining | Nucleus texture | nucleus - DAPI |
| Nucleus Hole | Hole pattern of nucleus staining | Nucleus texture | nucleus - DAPI |
| Nucleus Edge | Edge pattern of nucleus staining | Nucleus texture | nucleus - DAPI |
| Nucleus Ridge | Ridge pattern of nucleus staining | Nucleus texture | nucleus - DAPI |
| Nucleus Valley | Valley pattern of nucleus staining | Nucleus texture | nucleus - DAPI |
| Nucleus Saddle | Saddle pattern of nucleus staining | Nucleus texture | nucleus - DAPI |
| Nucleus Bright | Bright pattern of nucleus staining | Nucleus texture | nucleus - DAPI |
| Nucleus Dark | Dark pattern of nucleus staining | Nucleus texture | nucleus - DAPI |
| Cell (α-tubulin) Area | Cell area | Cell morphology | Cytoskeleton - α-tubulin |
| Cell (α-tubulin) Roundness | Cell roundness | Cell morphology | Cytoskeleton - α-tubulin |
| Cell (α-tubulin) Width | Cell width | Cell morphology | Cytoskeleton - α-tubulin |
| Cell (α-tubulin) Length | Cell length | Cell morphology | Cytoskeleton - α-tubulin |
| Cell (α-tubulin) Geometry | Ratio of cell width to length | Cell morphology | Cytoskeleton - α-tubulin |
| Cell (α-tubulin) Spot | Spot pattern of cell staining | Cell texture | Cytoskeleton - α-tubulin |
| Cell (α-tubulin) Hole | Hole pattern of cell staining | Cell texture | Cytoskeleton - α-tubulin |
| Cell (α-tubulin) Edge | Edge pattern of cell staining | Cell texture | Cytoskeleton - α-tubulin |
| Cell (α-tubulin) Ridge | Ridge pattern of cell staining | Cell texture | Cytoskeleton - α-tubulin |
| Cell (α-tubulin) Valley | Valley pattern of cell staining | Cell texture | Cytoskeleton - α-tubulin |
| Cell (α-tubulin) Saddle | Saddle pattern of cell staining | Cell texture | Cytoskeleton - α-tubulin |
| Cell (α-tubulin) Bright | Bright pattern of cell staining | Cell texture | Cytoskeleton - α-tubulin |
| Cell (α-tubulin) Dark | Dark pattern of cell staining | Cell texture | Cytoskeleton - α-tubulin |
| Cell (actin) Area | Cell area | Cell morphology | Cytoskeleton - F-actin |
| Cell (actin) Roundness | Cell roundness | Cell morphology | Cytoskeleton - F-actin |
| Cell (actin) Width | Cell width | Cell morphology | Cytoskeleton - F-actin |
| Cell (actin) Length | Cell length | Cell morphology | Cytoskeleton - F-actin |
| Cell (actin) Geometry | Ratio of cell width to length | Cell morphology | Cytoskeleton - F-actin |
| Cell (actin) Spot | Spot pattern of cell staining | Cell texture | Cytoskeleton - F-actin |
| Cell (actin) Hole | Hole pattern of cell staining | Cell texture | Cytoskeleton - F-actin |
| Cell (actin) Edge | Edge pattern of cell staining | Cell texture | Cytoskeleton - F-actin |
| Cell (actin) Ridge | Ridge pattern of cell staining | Cell texture | Cytoskeleton - F-actin |
| Cell (actin) Valley | Valley pattern of cell staining | Cell texture | Cytoskeleton - F-actin |
| Cell (actin) Saddle | Saddle pattern of cell staining | Cell texture | Cytoskeleton - F-actin |
| Cell (actin) Bright | Bright pattern of cell staining | Cell texture | Cytoskeleton - F-actin |
| Cell (actin) Dark | Dark pattern of cell staining | Cell texture | Cytoskeleton - F-actin |
