# Supplementary material for: Single‐cell high‐content imaging parameters predict functional phenotype of cultured human bone marrow stromal stem cells
Source: Stem Cells Transl Med. 2019 Nov 23;9(2):189–202. doi: 10.1002/sctm.19-0171 (PMC6988772; doi:10.1002/sctm.19-0171)
Supplement: Supplementary file 6 — Table S2: Supplementary information [file SCT3-9-189-s006.docx]

**Supplementary Table 2 Determination of predictive power**

| Determination of predictive power for **osteogenic** differentiation outcome using AIC analysis | | |
| --- | --- | --- |
| **Model** | **Value** | **R2** |
| m0 *(intercept)* | 522,06 |  |
| m1 *(nucleus geometry)* | 509,53 | 0.214 |
| m2 *(including: nucleus geometry, cell geometry  (α-tubulin), nucleus length and cell texture Spot)* | 513,68 | 0.195 |
| Determination of predictive power for **adipogenic** differentiation outcome using AIC analysis | | |
| **Model** | **Value** | **R2** |
| m0 *(intercept)* | 144,4 |  |
| m1 *(nucleus texture Hole)* | 142,9 | 0.044 |
| m2 *(including: nucleus texture Hole, nucleus geometry, cell geometry (actin) and cell texture Spot)* | 141,87 | 0.108 |
